# A finance and health collaboration to counter pandemic threats

**DOI:** 10.2471/BLT.23.291014

**Published:** 2024-03-18

**Authors:** Raymond Hutubessy, Ruchir Agarwal, Elizabeth Sarah Aryaputri

**Affiliations:** a Immunization, Vaccines and Biologicals Department, World Health Organization, Avenue Appia 20, 1211 Geneva 27, Switzerland.; b Mossavar-Rahmani Center for Business & Government, Harvard Kennedy School, Cambridge, United States of America.; c Ministry of Health, Jakarta, Indonesia.

The coronavirus disease 2019 (COVID-19) pandemic has highlighted the profound impact of global health emergencies on economic stability and human life. The International Monetary Fund projected a cumulative output loss of approximately 13.8 trillion United States dollars through 2024 due to the pandemic.^1^ Meanwhile, the World Health Organization (WHO) estimated excess deaths for 2020 and 2021 to be around 15 million, reflecting a significant loss of life as well as the broader societal toll of the pandemic.^2^ Even if serious pandemics occurred once or twice per century, the imperative for substantial investment in pandemic prevention, preparedness and response is clear, promising high returns in safeguarding lives and ensuring economic stability.

## Finance and health coordination

The COVID-19 pandemic has brought into focus the need for coordinated efforts between the finance and health sectors. Effective collaboration is crucial for optimal resource allocation, reducing economic fallout and prioritizing public health initiatives.

Pandemic preparedness policy comprises both health and economic policies, requiring nuanced policy-making that addresses the unique challenges of health emergencies.^3^ As we move forward, adopting a holistic approach is vital, necessitating a robust global framework that integrates financial and health strategies to create a unified response mechanism capable of addressing the complex challenges pandemics pose.

## Current state 

The Group of 20 (G20), an intergovernmental forum comprising 19 countries, the European Union and the African Union (AU), represents approximately 85% of the world’s gross domestic product, more than three quarters of world trade and two thirds of its population. This forum plays a pivotal role in addressing major issues related to global economic and financial stability. In October 2021, under the Italian presidency, the G20 established the G20 Joint Finance and Health Task Force, aiming to enhance dialogue and global cooperation on issues relating to pandemic prevention, preparedness and response.^4^ Under the Indonesian presidency, in November 2022 the Pandemic Fund was launched with the goal of financing critical investments in pandemic prevention, preparedness and response, focused on strengthening the capacities of low- and middle-income countries.^5^^,^^6^

The task force’s reach extended through India’s inclusive move to invite the AU as a permanent member during its G20 presidency in 2023, integrating wider perspectives and needs into its pandemic prevention, preparedness and response financing dialogue.^7^ Engaging regional organizations in 2023 such as the AU, Association of Southeast Asian Nations, and the Caribbean Community and Pacific Islands Forum, the task force has broadened its collaborative efforts, considerably increasing the number of countries involved.

During the Indonesia and India G20 presidencies, the task force established a foundation for improved global coordination in pandemic preparedness and response. The priorities of the Brazilian presidency for 2024 are under development.^8^

## The 2023 G20 survey

In 2023, the task force conducted a survey to better understand how finance and health sectors worked together among G20 members during the COVID-19 pandemic.^9^ This survey provided three important insights. 

First, universal recognition of the coordination need: G20 members unanimously acknowledged the need for robust coordination between finance and health sectors. Approximately 90% (23/26) of finance and health ministries reported overlapping mandates, emphasizing the importance of their collaborative efforts. 

Second, increased coordination levels: most G20 countries experienced an uptake in coordination levels during and after the pandemic. Notably, 92% (24/26) of these countries reported increased meeting frequencies during the crisis, a stark contrast to the 38% (10/26) before the pandemic. 

Third, diverse coordination approaches: the survey highlighted a variety of coordination methods, reflecting the diverse economic and health system landscapes across G20 countries. This diversity underscores the necessity for adaptable, country-specific strategies to ensure effective and versatile pandemic response measures.

## Future priorities

### Three finance and health priorities 

The deliberations and outcomes of the New Delhi G20 Leaders’ Declaration in September 2023 identified a set of forward-looking priorities.^7^ These priorities underscore a commitment to a more interconnected and resilient global health architecture for future pandemic preparedness and response. 

First, commitment to strengthen the global health architecture for pandemics. The New Delhi Declaration underscores the necessity for continued collaboration between finance and health ministries under the task force. The task force highlighted the importance of including key regional organizations to ensure low-income countries are represented. 

Second, commitment to the Framework on Economic Vulnerabilities and Risks and emphasis on the need for its continuous refinement. The framework assesses economic vulnerabilities and pandemic risks, considering the unique circumstances of each country. 

Third, commitment to engage in **further deliberations on how financing mechanisms could be optimized, better coordinated and when necessary, suitably enhanced to deploy the needed financing quickly and efficiently**.

### Refining the architecture

The task of refining the global health architecture for pandemics is an inclusive and iterative process, engaging a broad spectrum of stakeholders from various sectors and regions. This collaborative endeavour draws upon the expertise and resources of international partners, ensuring that the strategies developed are grounded in practicality and geared towards widespread applicability.

## Global health security

The COVID-19 pandemic has fundamentally transformed our perception of global health, positioning it as a critical element of both international security and prosperity. The interplay between finance and health has emerged as a crucial arena for developing effective pandemic management strategies. This collaboration, especially vital during health crises, necessitates strengthening to withstand future threats.

The lessons learnt from global, regional and national responses to the pandemic provide a blueprint for constructing solid defence mechanisms against future health emergencies. To advance global health security effectively, future work may need to consider three principles. 

First, adaptability: the nature of pandemics demands that our response strategies be as dynamic as the challenges they present. By crafting plans that are adaptable and applicable worldwide, we can ensure preparedness across diverse national landscapes, each with its unique health-care and economic contexts. Such a strategic approach promises a versatile defence, ready to evolve in the face of new threats. 

Second, openness: protecting global health requires enhanced coordination between and within countries – an endeavour that requires transparency and collective effort. This approach entails more than aligning on mutual goals; it necessitates active collaboration and better exchange of data and information. Aligning national policies within a shared vision for health security creates a solid front, crucial for immediate responses and the broader goal of establishing a comprehensive preparedness network globally. 

Third, localization: effective global coordination must have the capacity to resonate on a local scale. Through initiatives such as the Global Health Security Agenda and the Access to COVID-19 Tools Accelerator, we have observed the successful adaptation of global strategies to meet local requirements. Tailoring responses to local needs ensures that medical countermeasures are accessible to all nations, not just those with abundant resources.

Overall, the enhancement of finance and health coordination will promote health and economic well-being of communities worldwide. Committing to this partnership represents a dual investment in the health and economic resilience of societies. This holistic approach to global health security underscores the need for a unified strategy that leverages the strengths of both sectors, ensuring a robust response framework for future pandemics.
